# Measuring the activity of BioBrick promoters using an in vivo reference standard

**DOI:** 10.1186/1754-1611-3-4

**Published:** 2009-03-20

**Authors:** Jason R Kelly, Adam J Rubin, Joseph H Davis, Caroline M Ajo-Franklin, John Cumbers, Michael J Czar, Kim de Mora, Aaron L Glieberman, Dileep D Monie, Drew Endy

**Affiliations:** 1 Department of Biological Engineering, Massachusetts Institute of Technology, 77 Massachusetts Avenue, 68-580, Cambridge Massachusetts 02139, USA; 2 Department of Biology, Massachusetts Institute of Technology, 77 Massachusetts Avenue, Cambridge Massachusetts 02139, USA; 3 Molecular Foundry, Lawrence Berkeley National Laboratory, Berkeley, California 94720, USA; 4 Molecular Biology, Cell Biology, and Biochemistry, Brown University, Box G-W, Providence, Rhode Island, 02912, USA; 5 Virginia Bioinformatics Institute, Virginia Polytechnic and State University, Washington Street, MC0477, Blacksburg, VA 24061, USA; 6 Institute for Materials and Processes, The University of Edinburgh, King's Buildings, Mayfield Road, Edinburgh, Scotland EH9 3JL, UK; 7 Biomedical Engineering, Division of Engineering, Brown University, Providence, Rhode Island 02912, USA; 8 Department of Ophthalmology, Johns Hopkins University School of Medicine, 600 N Wolfe St, Baltimore, Maryland 21287, USA; 9 Profectus Biosciences, Inc., 6411 Beckley St, Baltimore, Maryland 21224, USA; 10 Department of Bioengineering, Stanford University, Stanford California 94305 USA

## Abstract

**Background:**

The engineering of many-component, synthetic biological systems is being made easier by the development of collections of reusable, standard biological parts. However, the complexity of biology makes it difficult to predict the extent to which such efforts will succeed. As a first practical example, the Registry of Standard Biological Parts started at MIT now maintains and distributes thousands of BioBrick™ standard biological parts. However, BioBrick parts are only standardized in terms of how individual parts are physically assembled into multi-component systems, and most parts remain uncharacterized. Standardized tools, techniques, and units of measurement are needed to facilitate the characterization and reuse of parts by independent researchers across many laboratories.

**Results:**

We found that the absolute activity of BioBrick promoters varies across experimental conditions and measurement instruments. We choose one promoter (BBa_J23101) to serve as an in vivo reference standard for promoter activity. We demonstrated that, by measuring the activity of promoters relative to BBa_J23101, we could reduce variation in reported promoter activity due to differences in test conditions and measurement instruments by ~50%. We defined a Relative Promoter Unit (RPU) in order to report promoter characterization data in compatible units and developed a measurement kit so that researchers might more easily adopt RPU as a standard unit for reporting promoter activity. We distributed a set of test promoters to multiple labs and found good agreement in the reported relative activities of promoters so measured. We also characterized the relative activities of a reference collection of BioBrick promoters in order to further support adoption of RPU-based measurement standards.

**Conclusion:**

Relative activity measurements based on an in vivoreference standard enables improved measurement of promoter activity given variation in measurement conditions and instruments. These improvements are sufficient to begin to support the measurement of promoter activities across many laboratories. Additional in vivo reference standards for other types of biological functions would seem likely to have similar utility, and could thus improve research on the design, production, and reuse of standard biological parts.

## Background

The engineering of many-component, synthetic biological systems is being made easier by the development of collections of reusable, standard biological parts [1-6]. Standardization of components has been instrumental in managing complexity in other engineering fields by helping engineers to reliably design and deploy systems comprised of combinations of parts [7]. However, it is an open question whether the overwhelming complexity of living systems will prevent biological engineers from fully achieving similar capabilities (below). To help answer this question, a Registry of Standard Biological Parts started at MIT now maintains and distributes thousands of BioBrick standard biological parts [8]. BioBrick parts provide the first popular example of standard biological parts. However, BioBrick parts are currently only standardized in terms of how individual parts are assembled into multi-component systems (that is, "physical composition") [1,9].

The utility of so-called standard biological parts would increase if the behavior of parts, both in isolation and in combination, were more predictable (that is, "functional composition")[9]. Prediction of behavior, in turn, depends on the initial designs and refinement of the parts themselves, the characterization of part functions, and the representation of part functions via abstract models (for related examples see [10-12]). Today, most BioBrick parts are directly derived from natural DNA sequences with only slight modifications to support at least one physical assembly standard, and many parts remain to be characterized. For example, fewer than 50 out of over 500 transcriptional promoters now available via the Registry have been characterized. Making matters worse, for the 50 characterized promoters, the methods of characterization are disparate and the resulting data incomparable. Shared and standardized approaches are needed in order to begin to address the challenge of characterizing promoters (and other types of standard biological parts) across a distributed community of biological engineers.

Making reliable and comparable in vivomeasurements of biological parts has proven challenging. For example, five different efforts to measure the abundances of proteins in the yeast pheromone mating response system, one of the best characterized eukaryotic signalling systems, produced reports for the numbers per cell (abundances) of key system proteins that vary over a factor of ~12 [Thomson TM, Benjamin KR, Bush A, Love T, Pincus D, Resnekov O, Yu R, Gordon A, Colman-Lerner A, Endy D, Brent R: Scaffold number in yeast signaling system sets tradeoff between system output and dynamic range. Molecular Systems Biology. *Unpublished*]. Such examples suggest that measurement of the state or activity of biological systems, whether natural or engineered, may be unlike past engineering experiences, in that the minor differences in experimental conditions (relative to what can be readily controlled for, below) may cause large changes in the properties being measured. Even if conditions could be controlled for, it has proven challenging for researchers to develop and adopt standard approaches for characterizing biological parts. For example, an analysis of 80 published papers in which researchers used beta-galactosidase (*β*-gal) activity as a measure of gene expression found that at least six different protocols were used to measure enzyme activity [13]. In addition, nearly all activities were reported in "Miller units" even though in several cases there were differences in the substrates used to quantify enzymatic activity (CPRG or ONPG), the experimental conditions (pH and temperature for the assay), and even the absolute units of the Miller unit (nmol/min or *μ*mol/min) [14]. Differences in conditions such as using either CPRG or ONPG as a substrate for enzymatic assays lead to incompatible results [15], and thus Miller units should generally not be considered comparable unless they have been calibrated against a common reference standard [13].

The challenge of making reliable in vivomeasurements of biological parts is further compounded by the need to measure many part properties indirectly via biological "measurement instruments" such as reporter proteins whose production can also be sensitive to experimental conditions. For example, *β*-gal activity can be used as an indirect measure of the behavior of a promoter, but the translation and activity of the *β*-gal protein is itself sensitive to experimental conditions such as temperature or choice of media. Since both the measurement instrument (*β*-gal) and the property being measured (promoter activity) are sensitive to measurement conditions (perhaps in differing ways) correcting for errors in measured promoter activity due to changes in conditions is more difficult. In theory such challenges could be addressed by strict adherence to standard measurement conditions. However, the adoption of standard measurement conditions in biological engineering is prevented by both practical constraints (as noted above) and also engineering constraints, such as culture or performance requirements that are specific to a particular biotechnology application. The overall situation is summed up nicely via the following quote: "There is no such thing as a standard (biological) component, because even a standard component works differently depending on the environment" [16].

Although the characterization of standard biological parts is challenging, lessons from the measurement of other types of physical objects are worth considering. For example, one approach to controlling for variation in the measured property of an object in response to changing experimental conditions is to collect data from which to develop a model that describes the relationship between the measured property and experimental conditions. As a specific example, models based on empirically determined coefficients of thermal expansion for common building materials (for example, Oak = 54E-6/K at 20°C; Stainless Steel = 17.3E-6/K at 20°C) are now sufficient to enable the reliable construction of structures across a range of environments [17]. However, given the complexity of living matter, the relationships between the measured properties of biological parts and experimental conditions may be difficult to determine (at first). Thus, a second lesson worth considering is the measurement of relative (or ratio) properties rather than absolute characteristics. A relative measure is the ratio of the measurement of some aspect of the object being characterized in comparison to a standard reference object that is measured under the same conditions. For example, early methods for the diagnosis of osteoporosis made use of a measure of spinal cord deformity that was based on the ratio of various length measurements of vertebra within an individual patient [18]. Doctors, by using a relative measurement for length, could account for variation in vertebra sizes between individuals of different body types or heights. As a second example, microarray experiments are frequently performed by co-hybridizing probes synthesized from both a reference and experimental RNA sample that have been labelled with different colors [19]; gene expression levels are then reported as the ratio of the experimental and reference intensities on each array spot. Thus, measurements made in relation to defined reference standards may provide an important first approach in characterizing the in vivoactivity of biological parts and, over time, could enable the collection of empirical data sufficient to support the development of models that describe the effect of varying conditions on part properties.

Here, we characterized the in vivoactivity of BioBrick promoters in order to evaluate if measuring relative activities might provide a useful initial framework for measuring the activity of standard biological parts across varying conditions. We chose to characterize promoters as a first example since they are ubiquitous in engineered biological systems, relatively well-understood, practically useful to biological engineers, and poorly characterized in the existing BioBrick collection [20,21]. We developed a system that allows indirect measurement of the activity of promoters via observation of the synthesis rate of Green Fluorescent Protein (GFP) encoded by mRNA transcribed from each promoter. Our system requires the use of a quantitative model that allows promoter activity to be estimated from observed rates of GFP synthesis (below). Using this approach we demonstrate that normalizing the apparent absolute activity of a promoter to a defined reference standard promoter can help account for variation in conditions that would otherwise lead to significant differences in reported measurements.

## Results

### Definitions and models for absolute promoter activity

Our first step in characterizing standard biological promoters was to choose the property or properties whose measure would best support the reuse of such parts by biological engineers. Since the primary use of promoters is to initiate transcription, we chose the rate of transcription initiation as the property to be measured. We next chose the promoter clearance rate as the specific property that best describes transcription initiation; we refer to this property as "promoter activity" throughout. In turn, we defined promoter activity as the number of RNA polymerase molecules that pass by (or clear) the final base pair of the promoter and continue along DNA as an elongation complex. We report promoter activity using the generic unit of "Polymerases Per Second," or PoPS, in place of the more traditional "promoter clearance rate" because reporting activity in PoPS allows promoters to be directly compared to other genetic parts whose functioning impacts elongating polymerases, such as transcription terminators [22]. Other properties of promoters such as the binding constant of RNA polymerase to the DNA encoding the promoter, or the secondary structure of the DNA were not considered; while such properties may be relevant to researchers who are studying or engineering new promoters, our focus here was to support researchers who are characterizing or reusing existing promoters.

Directly measuring PoPS in vivois challenging and, to our knowledge, has not yet been reported. However, by placing a promoter upstream of the coding sequence for green fluorescent protein (GFP) we could use the rate of GFP synthesis as an indirect measure of promoter activity. We could then use a quantitative model to relate observed GFP synthesis rates to promoter activities reported as PoPS.

We adopted a previously described ordinary differential equation (ODE) model of GFP expression from a constitutive promoter to relate GFP synthesis rates per cell to promoter activities [9,23]. We evaluated this ODE model at steady-state (Additional file 1) in order to determine the rates of successful mRNA initiation events per DNA copy of each promoter (PoPS^*SS*^) given observed GFP synthesis rates per cell ():

(1)

where *γ*_*M *_is the mRNA degradation rate, *a *is the GFP maturation rate, *γ*_*I *_is the degradation rate of immature GFP, *ρ *is the translation rate of immature GFP from mRNA, and *n *is the number of copies of the promoter in the cell.

### Variability due to equipment and conditions

**Figure 1:**
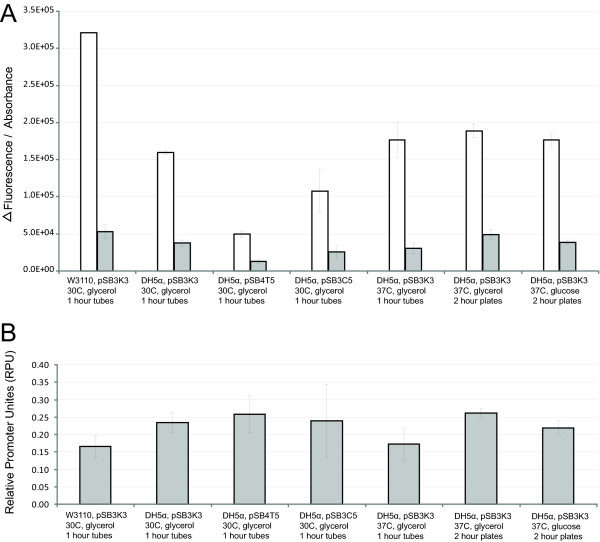
**Reference standards reduce variation in reported promoter activities under different measurement conditions**. We measured the activity of 2 promoters, J23101 (white columns) and J23150 (grey columns) under seven different measurement conditions and measurement instruments. We varied the media, temperature, cell strain, and plasmid copy number of the promoter test construct. (A) To estimate the per cell GFP synthesis rate we reported the change in fluorescence over a 1 hour period in exponential phase divided by the average absorbance during this period. The coefficient of variation of the GFP synthesis rates across the seven measurement approaches was 49% for J23101 and 39% for J23150. (B) We used the same data and divided the GFP synthesis rate of J23150 (grey bars) by that of J23101 (white bars) in order to calculate the relative promoter activity of J23150 in RPUs. The coefficient of variation of the relative promoter activity across the seven measurement approaches was only 17% suggesting that the relative promoter activity is less sensitive to conditions then absolute activity measured by per cell GFP synthesis rate.

We explored the sensitivity of our observable measure of promoter activity, GFP synthesis rates, to different measurement conditions and different measurement instruments. We estimated the per cell GFP synthesis rates of two promoters (BBa_J23101 and BBa_J23150) across seven different measurement conditions and instruments (Figure 1A). We estimated GFP synthesis rate by reporting the change in arbitrary fluorescence units per absorbance over a 1-hour period in log phase growth (Methods). We varied the experimental conditions by changing the cell strain (TOP10 or W3110), carbon source (glucose or glycerol), and temperature (30C or 37C) during growth. We found that the observed GFP synthesis rates were sensitive to the choice of strain (varying up to 2-fold) but insensitive to temperature and carbon source (within experimental error). We also varied the plasmid copy number and plasmid antibiotic resistance marker in order to explore how different genetic "measurement instruments" might impact the measured GFP synthesis rates. We found that GFP synthesis rates were sensitive to the plasmid copy number (varying up to 3-fold) and antibiotic resistance marker (varying up to 1.5-fold).

### Definition and models for relative promoter activities

We noted that the activity of promoters (for example, J23101 and J23150) measured across different conditions or with different instruments was correlated (Figure 1A). This correlation suggested that a measure of relative promoter activity might be less sensitive to varying experimental conditions or measurement instruments. To test this idea, we defined a new property – relative promoter activity – as the ratio of the absolute activity of a sample promoter, *φ*, relative to the absolute activity of a standard reference promoter, BBa_J23101, with both promoters measured under equivalent conditions and with the same measurement instrument. We reported relative promoter activities in a newly defined unit: Relative Promoter Units or RPUs. By our definition, a sample promoter with a relative activity of 1 RPU has activity equivalent to BBa_J23101.

An important consequence of considering a relative unit of measurement for reporting promoter activities is that many of the difficult-to-measure model parameters (Equation 1) that might change with changing environmental conditions can be cancelled when calculating relative promoter activities:

(2)

Thus, by substituting Eq. 1 into Eq. 2, we calculated promoter strengths in relative units of RPU and thereby eliminated many of the elementary parameters found in Eq. 1 via a cancellation of terms:

(3)

We then made four additional assumptions that further simplified Eq. 3. First, we assumed that GFP expressed from either the test promoter *φ *or the reference standard promoter has an equivalent maturation rate (*a*_*φ *_= *a*_*J*23101 _= *a*; given that the two promoters are measured under the same culture conditions). Second, since both promoters are carried on the same backbone plasmid, we assumed that each promoter is at the same average copy number (*n*_*φ *_= *n*_*J*23101_); while there are reported cases of promoter activity influencing the copy number of plasmids due to RNA polymerases transcribing through the plasmid origin of replication [24], a transcription terminator (BBa_B0015) downstream of our test construct's GFP coding sequence as well as the transcription terminators flanking the BioBrick cloning site [25] should largely prevent differences in promoter activity from impacting plasmid copy number. Third, since the promoters tested here have been standardized to have identical transcription initiation sites (predicted) and identical sequences downstream of the initiation site (Additional file 1) we expected that each promoter produces the same mRNA sequence [26]. Since the transcribed mRNAs are expected to be identical we assumed that their mRNA degradation rates are equivalent (*γ*_*M*, *φ *_= *γ*_*M*, *J*23101_) and that the translation rates of immature GFP from mRNA are also equivalent (*ρ*_*φ *_= *ρ*_*J*23101_); while mRNA degradation is also a function of dilution due to cell growth, the dilution rate is negligible relative to typical rates of active mRNA degradation in *E. coli *[27]. Finally, we assumed that immature GFP is stable so that protein degradation is negligible compared to dilution due to cell growth (*γ*_*I*, *φ *_= *μ*_*φ *_and *γ*_*I*, *J*23101 _= *μ*_*J*23101_, where *μ *is the cellular growth rate). Following the above assumptions, we simplified Eq. 3 to:

(4)

We further simplified Eq.4 by noting that:

(5)

For example, we measured the growth rates of cells in our experiments to determine if the difference between the growth rates of cells containing the promoter test construct (*μ*_*ϕ*_) and cells containing the reference standard construct (*μ*_*J*23101_) is negligible compared to the maturation rate of GFP (that is, |*μ*_*ϕ *_- *μ*_*J*23101_| ≪ *a*). The cellular growth rates varied depending on the promoter being tested as well as on the experimental conditions: the fastest growth rate was observed in cells containing the BBa_J23113 promoter test construct grown in M9+glucose (*μ *= 0.9 hr^-1^), and the slowest growth rate was observed in cells containing the BBa_R0040 promoter test construct grown in M9+glycerol (*μ *= 0.5 hr^-1^). The maturation rate of the GFP variant used in the GFP reporter device (BBa_E0040) has been measured previously as *a *= 6.48 hr^-1^[9]. Based on the worst-case assumption that cells containing the promoter test construct are the fastest growing cells (*μ*_*ϕ *_= 0.9 hr^-1^), and that cells containing the reference standard construct are the slowest growing cells (*μ*_*J*23101 _= 0.5 hr^-1^) then:

(6)

Therefore, we assumed that the difference between the growth rates of cells containing the promoter test construct (*μ*_*ϕ*_) and cells containing the reference standard construct (*μ*_*J*23101_) is negligible compared to the maturation rate of GFP, allowing Eq. 4 to be combined with Eq. 5 yielding:

(7)

Taken together, by reporting promoter activity relative to a reference standard promoter (BBa_J23101) and choosing promoters with identical transcription initiation sites and identical sequences downstream of the initiation sites, researchers can quickly report measured relative promoter activities in compatible units without having to independently measure GFP maturation rates, mRNA degradation rates, protein production rates, or plasmid copy number for their specific experimental setup. (We detail the precise numerical sensitivity of the quantitative model to each of the above assumptions in Additional file 1).

We converted GFP synthesis rates measured across 7 different conditions and instruments (Figure 1A; Coefficient of variation (CV) of the measurements is 39.1%) to relative promoter activity in RPUs (Figure 1B; CV of the measurements is 17.5%). We noted that the coefficient of variation in promoter activity was reduced by approximately half when converted to RPUs from GFP synthesis rates. This reduction in variation suggests that relative promoter activity might be a useful property for characterizing promoters. However, care should be taken to note that while relative promoter activities remain fairly constant across some range of conditions, absolute promoter activities vary widely across these same conditions. Stated differently, a promoter that has an equivalent relative activity across multiple conditions might not produce equal absolute activity (as measured in PoPS) across the same conditions (please see Discussion).

### Laboratory-laboratory variation

**Figure 2:**
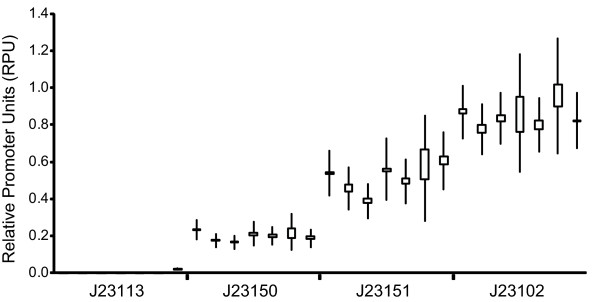
**Reference standards and units allow independent labs to make sharable measurements**. Each laboratory followed the same measurement procedure, measuring relative promoter activities based on GFP concentration measured via a flow cytometer. Measurements were taken in triplicate; the boxes show the highest and lowest measured relative promoter activities and the whiskers show the 95% confidence interval of the mean of the activities. The large range in the 95% confidence interval (extending beyond the highest and lowest measured activities) is partially a function of the small number of replicates (three) that were conducted by each laboratory. The activity of BBa_J23113 was equivalent to the negative control within error for all but one of the laboratories. The measured activities of the other three promoters were fairly consistent across laboratories with less than a 2-fold range of activities measured for each promoter across all labs (BBa_J23150: 0.14 – 0.23; BBa_J23150: 0.38 – 0.606; BBa_J23103: 0.77 – 0.96).

Our initial success in characterizing relative promoter activity across different conditions and measurement instruments suggested a practical test. Specifically, we sought to determine whether multiple laboratories could work together to characterize promoters. To do this, we distributed a "reference promoter set" comprised of four strains, each containing one promoter test construct (BBa_J23113, BBa_J23150, BBa_J23151, or BBa_J23102) to researchers in six independent laboratories. Each researcher then measured the activity of the four promoters following a five-step procedure: (1) three independent cultures were grown from single colonies for each of the four promoters, (2) cells were collected in exponential phase, (3) GFP concentration per cell was measured using a flow cytometer, (4) flow cytometer data was gated based on forward and side scatter and the negative control, and (5) the geometric mean of the per cell fluorescence in the population was reported for each culture (Methods). We made no efforts to standardize the equipment (flow cytometers) or equipment settings beyond asking researchers to use typical settings for measuring GFP and by providing each lab with an example plot to guide gating of the flow cytometry data based on forward scatter, side scatter, and fluorescence [28]. As expected, there were slight differences in how the protocol was conducted in each laboratory, such as different culture conditions (rollers or shakers) and growth time. Since the measurements were reported in common units of RPUs we are able to compare the results of the interlaboratory promoter activity measurements directly (Figure 2). The mean promoter activity measured by each lab is relatively consistent across all laboratories with less than a 2-fold range of activities (min-max) across all measured promoters (BBa_J23150: 0.14–0.23 RPU; BBa_J23150: 0.38–0.61 RPU; BBa_J23103: 0.77–0.96 RPU). The activity of the weakest promoter, BBa_J23113, was equivalent to the negative control within error for all but one of the laboratories. These results suggest that relative promoter activity is an effective metric for making comparable measurements across multiple laboratories. Finally, we determined the coefficients of variation of the measured promoter activities across all labs to be 17.2%, 17.1%, and 8.5% for BBa_J23150, BBa_J23151, and BBa_J23101, respectively, setting a baseline for future improvements to the measurement kit and methods.

### Community-based measurement of promoter collections

**Figure 3:**
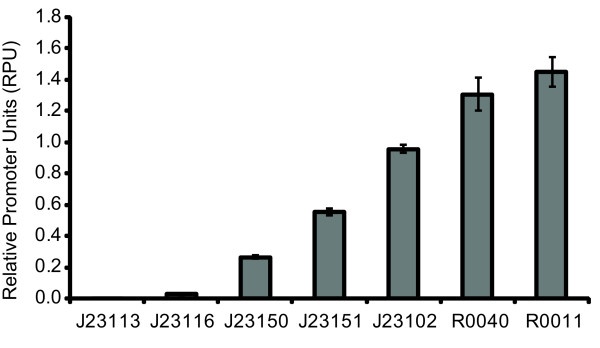
**Promoter collections can be readily characterized via Relative Promoter Units (RPUs)**. The five promoters labeled J23### are from a constitutive promoter library and R0040 and R0011 are tet- and lac-repressible promoters, respectively. The activity of the promoters was measured in relative promoter units (RPUs). This collection of promoter may itself be useful for tuning gene expression in engineered systems. The error bars represent the 95% confidence interval of the mean based on nine replicates.

Given that many laboratories could coordinate their measurement of promoter activities, we sought to prepare tools that would facilitate the widespread adoption of relative promoter activity measurements. To do this we first measured the relative activities of a set of seven representative promoters obtained from the Registry of Standard Biological Parts (Figure 3) [8]. These promoters included members of a constitutive promoter library (BBa_J23100 – BBa_J23119, constructed by JC Anderson) as well as the commonly used Tet repressor (BBa_R0040) and Lac repressor (BBa_R0011) regulated promoters [29]. The regulated promoters were tested in the absence of their cognate repressor proteins. Such libraries of characterized promoters have been shown to be valuable to researchers for tuning biochemical networks to optimize the synthesis of products of interest [20,30]. We measured the relative promoter activities by calculating the steady-state GFP synthesis rates (Methods) and converting these rates to RPUs. Nine independent clones were characterized across three separate experimental runs for each promoter tested. The promoters ranged in activity from 0.026 ± 0.003 to 1.45 ± 0.095 RPUs (uncertainties represent 95% confidence interval of the mean). The GFP expression level from one promoter (BBa_J23113) was statistically equivalent within measurement error to the expression level of the negative control (TOP10).

To further support community-based standardized measurement of promoter activities, we developed a first generation measurement kit for characterizing the relative activity of BioBrick promoters in RPUs. Our overarching objective for the kits was to enable independent researchers to make comparable measurements of relative promoter activity in standard units. We developed instructions and a parts list for the promoter measurement kit (Additional file 1 [Supp Box1 and Supp Table1]). The promoter measurement kit contains measurement "instruments" such as a green fluorescent protein (GFP) reporter device (BBa_E0240) and backbone plasmid (pSB3K3), as well as a recommended *E. coli *strain (TOP10). The reference promoter (BBa_J23101) was inserted upstream of the GFP reporter device (BBa_E0240) and included in the kit as the reference standard construct (BBa_I20260). In order to measure the activity of a user-specified promoter, kit users assemble the user-specified promoter upstream of the GFP reporter device and insert this combined part into the backbone plasmid to form the promoter test construct. The process for inserting a promoter upstream of the GFP reporter device is based on three-antibiotic BioBrick standard assembly [25], and is outlined in the instructions included with the kit (Additional file 1 [Supplementary Box 1]).

## Discussion

We found the absolute activity of promoters to vary under different experimental conditions and when using different measurement instruments. We chose a promoter (BBa_J23101) to serve as a reference standard and demonstrated that by measuring relative promoter activity (activity of a sample promoter divided by activity of the reference standard promoter BBa_J23101, measured under the same conditions) we could reduce reported variation in measured promoter activity across differing experimental conditions and equipment. We defined the Relative Promoter Unit (RPU) in order to enable researchers to report promoter characterization results in compatible units, and developed a measurement kit in order to more easily allow researchers to adopt an RPU-based measurement approach. We distributed a test set of 4 promoters to 7 independent labs and found good agreement in the measured relative promoter activities across the test set. Finally, we characterized the relative promoter activity of 7 BioBrick promoters in order to bootstrap a collection of promoters measured according to our initial RPU reference standard.

### Absolute and relative promoter activities

The absolute activity of a promoter is defined by the number of elongating polymerases per second (PoPS) exiting the promoter. The same promoter under different environmental conditions can have widely varying absolute promoter activities (Figure 1A). Moreover, it is challenging to relate an indirect measure of absolute promoter activity, such as per cell GFP synthesis rates, to PoPS since any variation across conditions in the functioning of the genetic measurement instrument may not be well correlated with variation in promoter activity across these same conditions. In contrast, the relative activity of a promoter is defined as the ratio of the absolute activity of the promoter to the absolute activity of a reference standard promoter measured under the same conditions and with the same measurement instrument. We found that the relative activity of a promoter will remain fairly constant across a practical range of conditions (Figure 1B).

Relative promoter activity as reported in RPUs enables the ranking of the activities of promoters but does not, by itself, provide information about the absolute activity of the promoter under particular conditions. For example, if the relative activity of a sample promoter remains constant across several conditions, it is not necessarily the case that the promoter is producing equal numbers of mRNA transcripts in each condition, only that the promoter activity is remaining proportional to the reference standard across the conditions. Nonetheless, relative promoter activity is a valuable property to measure and report since promoters can be rank ordered by relative promoter activity even if they were characterized by different researchers or across different environments (Additional file 1 [Supplementary Table 4]). Furthermore, if both the relative and absolute activities of a promoter are measured, then a conversion factor can be established that defines the relationship between relative and absolute promoter activity under specific measurement conditions. For example, the reference standard promoter J23101, which has a relative activity of 1 RPU, has an estimated absolute activity of ~0.03 PoPS under specific conditions (Additional file 1). Therefore, the promoter J23151, which has a relative activity of approximately 0.5 RPU under these same conditions, would be predicted to have an absolute activity of ~0.015 PoPS. As more absolute measurements are made, an expanded set of conversion factors (or functions) could be developed, allowing for improved estimates of absolute promoter activities across a wider range of measurement conditions.

### Interlaboratory measurement of promoter activity

Measurement of in vivopromoter activities across laboratories is challenging due to the sensitivity of results to both experimental conditions and measurement instruments (Figure 1), as well as the lack of shared reference standards [13]. We demonstrated here that by using a relative promoter activity to characterize promoter strength based on a shared reference standard, seven independent laboratories could make comparable measurements of three promoters (Figure 2). We expect that future improvements to the recommended measurement techniques and measurement kit components could further reduce the variation in measurements across laboratories, with the results reported here providing a practical baseline for judging proposed improvements.

### Measurement procedures

The reference promoter BBa_J23101 and relative promoter activity measured in RPUs provide a shared platform for researchers to evaluate different measurement procedures. We deliberately have not advocated a single measurement procedure, and there should be many acceptable procedures for characterizing promoters in RPUs, just as units for length or mass are not tied to a single measurement approach. The choice of the best measurement procedure will be influenced by the particular group making the measurements. For example, laboratories without access to equipment for capturing high-throughput single-cell measurements of fluorescence might opt for a bulk fluorescence measurement using a fluorimeter. Other groups might prefer to obtain single-cell measurements using quantitative microscopy or flow cytometry, or to capture a time-course of fluorescence measurements from a growing culture. As different measurement procedures are likely to have merits within different communities we expect that a number of procedures will be established. As an example, for the community of undergraduate teams using the promoter measurement kit during the International Genetically Engineered Machines (iGEM) competition, we have suggested a measurement protocol that can be easily carried out by novice researchers and that only requires two absorbance and bulk fluorescence measurements (Additional file 1 [Supplementary Box 2]) [31].

### Engineering with characterized promoters

We anticipate that both absolute and relative promoter measurements will be useful in engineering genetic networks, however it is unclear to what extent one approach might be preferred over the other (presuming both types of measurements could be readily obtained). For example, we can imagine an engineering design framework in which the absolute activities of promoters and other functional genetic elements are tracked explicitly, in order to support detailed modeling and analysis of issues such as the absolute "load" placed on a host cell via recombinant gene expression. Such an ability seems likely to become more important as many-component engineered biological systems are attempted (dozens to hundreds of gene products), in which the absolute expression levels of individual genes must be well managed and might be kept low compared to the high-expression, protein production systems typically used today. However, we can also imagine a competing or complementary engineering framework, based on the idea that cells already provide self-adapting and robust environments within which the absolute activities of genetic elements such as promoters are finely regulated by overall environmental or culture conditions [32]. In such a framework, relative measurements of promoter activities may be both easier to obtain and more relevant. Many natural biological systems already follow this model, and are robust to the absolute properties of components so long as the relative relationships between subparts are maintained. For example, developmental body plans may vary in size with individual organisms having different overall sizes, however the ratio of the sizes of individual bones or organs to overall body mass is often tightly maintained [33].

### Standard promoter definition

The promoters tested here were practically standardized to have identical transcription initiation sites (predicted) and identical sequences downstream of the initiation site (Additional file 1). Thus, we expect that the mRNA expressed by each of the tested promoters is identical to the mRNA produced by the reference standard promoter, and that we can cancel the mRNA degradation rate and translation rate of immature GFP from mRNA terms in simplifying the model relating GFP synthesis rates to RPUs. This simplification allows the activity of promoters to be reported in comparable units (RPUs) without needing to directly measure mRNA levels. Going forward, an expanded definition for standard BioBrick promoters could be developed in order to ensure that all promoters share the same transcription start position and a fixed sequence downstream of the transcription start site (that is, 5' mRNA UTR). All promoters that adhered to such a standard could then be reliably measured using the kits described here, or via future kits based on gene expression reporters that adhered to any new standard, without the need for promoter-specific mRNA quantitation.

### Distribution, use, and improvement of standardized measurement kits

Shared measurement tools and reference standards become more useful as they are broadly adopted. To facilitate such adoption, the Registry of Standard Biological Parts now includes our promoter measurement kit and reference standard in the annual distribution of BioBrick parts. We also created a website in order to support the reporting and sharing of promoter activity measurements . This website contains instructions for use of the kit and summarizes previously characterized promoters. Finally, to enable discussion of proposed improvements to the kit and reference standard, and also the development of new kits and reference standards, we are supporting an open discussion of technical standards in synthetic biology .

## Conclusion

Standard tools, techniques, and units for measurement are needed for a distributed community of biological engineers to independently characterize and share biological parts. We have defined a shared unit for measuring relative promoter activity (Reference Promoter Units, RPUs) and demonstrated that relative promoter activity can address some of the challenges in measurement across labs due to varying experimental conditions and measurement instruments. We developed a first-generation measurement kit for BioBrick promoters, and are freely distributing the kit via the Registry of Standard Biological Parts. Having demonstrated the feasibility and ease of use of the kit, we hope to encourage a community of users to adopt and improve these measurement tools and reference standard in order to characterize promoters via a comparable and common unit, the RPU. We expect that the shared experiences of biological engineers using common measurement tools and standards will help to identify new engineering challenges in improving the reliability and reuse of standard biological parts.

## Methods

### Strains and media

All measurement experiments and cloning were performed in *E. coli *TOP10 (Invitrogen) or W3110. Supplemented M9 minimal medium (M9 salts, 1 mM thiamine hydrochloride, 0.2% casamino acids, 0.1 M MgSO_4_, 0.5 M CaCl_2_) was used for all measurement experiments with either glycerol (0.4%) or glucose (0.4%) added as a carbon source and kanamycin (20 *μ*g/ml) antibiotic added where appropriate. All oligonucleotides were purchased from Invitrogen and DNA modifying enzymes were purchased from New England Biolabs.

### Promoter measurement kit contents

Sequences for all BioBrick plasmids (denoted pSB***) and BioBrick parts (denoted BBa_####) are available through the Registry of Standard Biological Parts [8]. pSB3K3 contains a p15A origin of replication (copy number 10–12) and the kanamycin resistance marker, pSB4T5 contains the pSC101 origin of replication (copy number ~5) and the tetracycline resistance marker, and pSB3C5 contains the p15A origin of replication and the chloramphenicol resistance marker. Physical copies of the plasmids and parts are also available from the Registry via the annual Registry parts distribution. The details of the promoter measurement kit contents are described in Supplementary Box 1 and Supplementary Table 1 (see Additional file 1). The sequences for the preparative primers used to amplify pSB3K3 to generate backbone plasmid are: TACTAGTAGCGGCCGCTGCAG (forward primer) and CTCTAGAAGCGGCCGCGAATTC (reverse primer).

### Assembly of test constructs

We built promoters by annealing synthesized oligonucleotides. The oligonucleotides were ordered with 5' phosphates and designed to leave an EcoRI overhang on the 5' end and a SpeI overhand on the 3' end so they could be used in subsequent ligation reactions without an intermediate restriction digest step. We inserted seven promoters: BBa_J23113, BBa_J23116, BBa_J23150, BBa_J23151, BBa_J23102, BBa_R0040, and BBa_R0011 into the promoter test construct and transformed into TOP10 according to the process outlined in Supplementary Box 1 (see Additional file 1). We found the optimal concentration of DNA for each of the three components in the ligation reaction (pSB3K3, BBa_E0240 or BBa_I13401, and the test promoter) was approximately 10 ng per uL. More detailed protocols and troubleshooting can be found at . In the process of construction we found mutations in two of the promoters that we attribute to errors in the synthesis of the oligonucleotides that were annealed to construct the promoters. The two promoters were functional so we included them as additional members of the collection (BBa_J23150 and BBa_J23151). The method of part assembly described here is based on the three-antibiotic BioBrick standard assembly method [25].

### Assay of promoter collection

The protocol described here will be referred to as the "original" protocol throughout the methods section and describes the measurement procedure used to characterize the set of seven promoters (Figure 3). For each promoter construct three 17 mm test tubes containing 5 ml of pre-warmed (37°C) supplemented M9 medium with kanamycin (20 *μ*g/ml) were inoculated from single colonies of TOP10-DH5*α *containing the promoter test construct on the pSB3K3 vector backbone. Cultures were grown in 17 mm test tubes for approximately 20 hrs at 37°C with spinning at 70 rpm. We then diluted the cultures 1:100 into 5 ml of pre-warmed fresh media and the cultures were grown for approximately four hours under the previous conditions (17 mm tubes, 37°C, spinning at 70 rpm). After four hours, we measured the OD600 of a 500 *μ*l aliquot from each culture on a WPA Biowave Spectrophotometer. Based on this OD measurement, the cultures were diluted to the same OD (0.07) in 5 ml of pre-warmed fresh media and grown for one hour at 37°C. We then transferred three 200 *μ*l aliquots from each culture into a flat-bottomed 96 well plate (Cellstar Uclear bottom, Greiner). We incubated the plate in a Wallac Victor3 multi-well fluorimeter (Perkin Elmer) at 37°C and assayed with an automatically repeating protocol of absorbance measurements (600 nm absorbance filter, 0.1 second counting time through 5 mm of fluid), fluorescence measurements (485 nm excitation filter, 525 nm emission filter, 0.1 seconds, CW lamp energy 12901 units), and shaking (3 mm, linear, normal speed, 15 seconds).

Background absorbance was determined by measuring wells containing only media. Background fluorescence was determined at different ODs from the fluorescence of TOP10 cells without a GFP expressing vector [34]. After background subtraction, time-series fluorescence (*F*) and absorbance (*ABS*) measurements were used to calculate the ratio of the rates of GFP synthesis for the promoter test construct and the reference standard construct. Measurements were taken from an approximately 30 min period in midexponential growth [35] (Additional file 1 [Supplementary Materials Figure 1 & 2]). For example:

(9)

Since we are calculating a ratio of the GFP synthesis rates we do not need to determine each rate in absolute units of GFP per second per cell, rather we can use the background-subtracted fluorescence (*F*) that is proportional to the number of GFP molecules and the background-subtracted absorbance (*ABS*) that is proportional to the number of cells in the culture to calculate the ratio of GFP synthesis rates [9,36].

### Assay of different measurement conditions

We measured the promoter activity of two promoters (BBa_J23101 and BBa_J23150) under seven different measurement procedures. The first of the seven procedures was identical to the "original" protocol described above for measuring the 7-member promoter collection except it was conducted at 30°C in the strain W3110 with pSB3K3 as the vector backbone for the promoter test construct. The second procedure was identical to the original except it was conducted at 30°C. The third procedure was identical to the original except that it was conducted at 30°C and pSB4T5 was used as the vector backbone. The fourth procedure was identical to the original except that it was conducted at 30°C and used pSB3C5 as the vector backbone. The fifth procedure was identical to the original. The sixth procedure was identical to the original except that instead of the second dilution into tubes followed by 1 hour of growth, the cells were diluted into 96 well plates and incubated for two hours before we started taking measurements. The seventh procedure was identical to the sixth except glucose was used instead of glycerol as the carbon source.

### Assay of inter-laboratory variability

We distributed a set of four promoters (BBa_J23113, BBa_J23150, BBa_J23151, and BBa_J23102) to six laboratories to take independent measurements of promoter activity. The protocol each lab conducted was identical to the original protocol described, except that the cells were harvested after the first 1:100 dilution and 4 hours of growth (there was no second dilution step). The cells were then spun down, resuspended in PBS, and the fluorescence per cell was measured using a flow cytometer. The measurement equipment used (cytometer model, laser, emission filter) varied between the laboratories (see Additional file 1 [Supplementary Table 2]).

For all other experiments we measured RPUs from the GFP synthesis rates as described in Equation 9. However, for the inter-laboratory experiments we are unable to measure the GFP synthesis rates because these rates require a time series to calculate (*dG*/*dt *in Eq. 9) and we only requested a single time point, however we can use this single time point to find the background-subtracted per cell fluorescence at steady-state ([*F*]). The flow cytometer measures fluorescence per cell directly, thus (*F*) is calculated by taking the geometric mean of the population fluorescence per cell. We related the per cell GFP concentration ([*G*]) to RPUs by using a model described previously [23] (derivation in Additional file 1):

(10)

*μ*_*φ*_/*μ*_*J*23101 _is a correction term based on differences in growth rate. Changes in the growth rate effect per cell GFP accumulation since loss of GFP per cell is largely due to dilution. Since we are calculating a ratio of the per cell GFP concentrations we do not need to determine each rate in absolute units of GFP molecules per cell, rather we can use the background-subtracted per cell fluorescence ([*F*]) that is proportional to the number of GFP molecules per cell to calculate the ratio of GFP concentrations.

After background correction, the per cell fluorescence ([*F*]) was determined for each promoter and activities in RPUs were calculated using Eq. 10. We applied the growth rates measured previously (Additional file 1 [Supplementary Table 3]) across all laboratories when calculating RPUs, rather than requesting individual laboratories to measure growth rates. This approximation likely increased the variability in the promoter activity measurements across laboratories, as growth rates vary between laboratories due to differences in culture conditions and media.

## Competing interests

The authors declare that they have no competing interests.

## Authors' contributions

JK, AR, and JD designed and conducted all experiments except those done at other laboratories. JK, KD, and CA designed the multi-institution experiments which were conducted by AR, CA, JC, MC, KD, AG, and DM. JK and DE conceived of the study and wrote the manuscript. All authors read and approved the final manuscript.

## Supplementary Material

Additional file 1 Supplementary material. Various supplemental materials Click here for file
