# Supplementary material for: Measuring the activity of BioBrick promoters using an in vivo reference standard
Source: J Biol Eng. 2009 Mar 20;3:4. doi: 10.1186/1754-1611-3-4 (PMC2683166; doi:10.1186/1754-1611-3-4)
Supplement: Additional file 1 — Supplementary material. Various supplemental materials [file 1754-1611-3-4-S1.doc]

Supplementary material

Table of contents

[Modeling 3](#__RefHeading___Toc222200859)

[Derivation of the relationships between absolute promoter activity and GFP synthesis rate or GFP concentration 3](#__RefHeading___Toc222200860)

[Derivation of promoter activity in RPUs as a function of GFP synthesis rate 3](#__RefHeading___Toc222200861)

[Derivation of promoter activity in RPUs as a function of GFP concentration 3](#__RefHeading___Toc222200862)

[Promoter measurement kit design and construction 3](#__RefHeading___Toc222200863)

[Alternate designs for the promoter measurement kit 3](#__RefHeading___Toc222200864)

[Transcription start site prediction 3](#__RefHeading___Toc222200865)

[Calculating the relationship between PoPS and RPUs 3](#__RefHeading___Toc222200866)

[Parameter sensitivity 3](#__RefHeading___Toc222200867)

[Supplementary references 3](#__RefHeading___Toc222200868)

[Supplementary figures 3](#__RefHeading___Toc222200869)

[Supplementary Figure 1 - Example of typical growth curves 3](#__RefHeading___Toc222200870)

[Supplementary Figure 2 - Example of GFP synthesis rate time series 3](#__RefHeading___Toc222200871)

[Supplementary Figure 3 - Parameter sensitivity 3](#__RefHeading___Toc222200872)

[Supplementary Box 1 - Instructions for inserting a promoter into the promoter measurement kit and measuring the promoter activity in Relative Promoter Units (RPUs). 3](#__RefHeading___Toc222200873)

[Supplementary Box 2 - Instructions for measuring promoter activity in Relative Promoter Units (RPUs) provided to participants in the International Genetically Engineered Machines (iGEM) competition. 3](#__RefHeading___Toc222200874)

[Supplementary tables 3](#__RefHeading___Toc222200875)

[Supplementary Table 1 - Components of promoter measurement kit 3](#__RefHeading___Toc222200876)

[Supplementary Table 2 - Listing of the flow cytometer equipment used at the seven laboratories that participated in the inter-laboratory variability study. 3](#__RefHeading___Toc222200877)

[Supplementary Table 3 - Growth rates of cells containing the 4 promoter test constructs that were used in the inter-laboratory study measured relative to the reference standard. 3](#__RefHeading___Toc222200878)

[Supplementary Table 4 - Overview of relative and absolute promoter activities 3](#__RefHeading___Toc222200879)

# Modeling

## Derivation of the relationships between absolute promoter activity and GFP synthesis rate or GFP concentration

In order to calculate the absolute promoter activity (
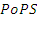
) from either the per cell GFP synthesis rate or the per cell GFP concentration at steady state for a promoter measured using the measurement kit, we extended a previously described ODE model [1] for GFP expression from a constitutive promoter. Our model is shown here:


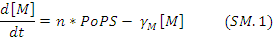


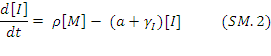


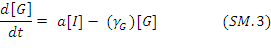


We also define the per cell mature GFP synthesis term specifically as:


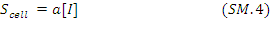


Where
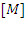
 is the concentration of mRNA,
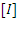
 is the concentration of immature GFP,
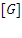
 is the concentration of mature GFP,
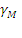
is the mRNA degradation rate,
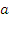
 is the GFP maturation rate,
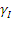
 is the degradation rate of immature GFP,
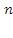
 is the copy number of the plasmid containing the promoter,
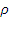
 is the rate of synthesis of immature GFP in absolute units of protein per second per mRNA, and
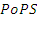
 is the rate of mRNA synthesis in absolute units of successful mRNA initiation events per second per DNA copy of the promoter.

These equations can be used to establish two relationships where promoter activity (
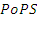
) is specified as a function of the experimentally measurable terms in the model (GFP synthesis rate, or
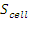
and GFP concentration, or
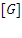
). Each of these relationships can be determined easily by assuming the system is at steady state, and thus
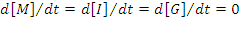
. The results of the two cases are:


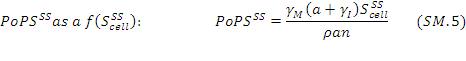


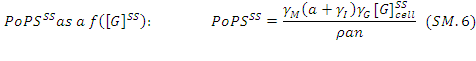


## Derivation of promoter activity in RPUs as a function of GFP synthesis rate

As a reminder, we define RPUs as a relative measurement in terms of the activity of a test promoter φ and the activity of the reference standard promoter BBa_J23101 as:


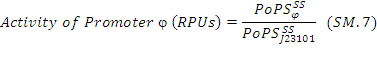


We derived promoter activity in RPUs as a function of GFP synthesis rate in the main text and will only review it briefly here. In the main text we use Eq. SM.5 along with a number of assumptions of equivalent rates in the promoter test construct and the reference standard construct to derive the activity of promoter φ in RPUs as a function of the measured GFP synthesis rates (
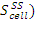
:


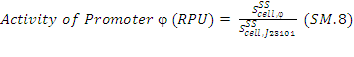


## Derivation of promoter activity in RPUs as a function of GFP concentration

We can derive promoter activity in RPUs as a function of per cell GFP concentration by combining Eq. SM.7 and SM.6 to yield:


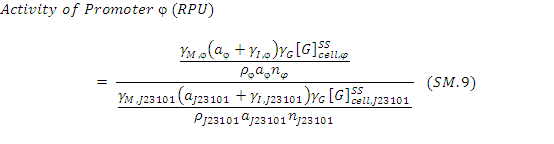


We again make the same assumptions described in the main text to equate several parameters for the promoter test construct and the reference standard construct, allowing for cancelling of terms. The only additional assumption that needs to be made is that mature GFP is stable so that protein degradation is negligible compared to dilution due to cellular growth (
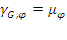
 and
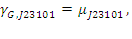
where
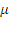
 is the cellular growth rate). This assumption is reasonable as the GFP used here does not have a degradation tag. These assumptions allow us to simplify Eq. SM.9 and find a simple relationship relating the activity of promoter φ in RPUs as a function of the measured GFP concentrations (
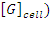
, the growth rate of the cells containing the promoter test construct (
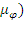
, and the growth rate of the cells containing the reference standard (
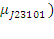
.


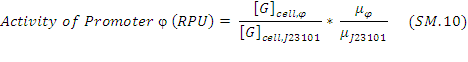


# Promoter measurement kit design and construction

We had four goals in designing the promoter measurement kit. First, we wanted the DNA manipulation necessary for assembling the promoter test construct to be simple and reliable so that the kits could be adopted as widely as possible. Second, we wanted to choose a reporter of promoter activity that could be detected under a wide range of possible measurement conditions. For example, researchers might want to characterize parts at different cellular growth phases, under different culture conditions, with different instruments, or at different resolutions (for example, single-cell or in bulk culture). Third, we wanted reference standards for promoter activity that were similar to the activities of other promoters commonly used in engineered biological systems so that our measurement kits would be relevant to many biological engineers. Fourth, we wanted the characterization of a promoter via the measurement kits to provide an accurate estimate of the performance of the promoter when reused in any engineered system built using standard BioBrick DNA assembly methods.

To meet our first design goal we needed a reliable method to insert a promoter upstream of GFP in our backbone plasmid, pSB3K3. We designed the DNA parts included in the kit (BBa_E0240 and pSB3K3) for assembly via three-antibiotic BioBrick standard assembly methods [2]. These assembly methods have proven simple enough to be reliably implemented by many first-time researchers in the annual International Genetically Engineered Machines (iGEM) Competition [3]. We chose *E.coli* TOP10 (Invitrogen) as the testing strain included in the kit due to the high transformation efficiency of the strain.

To meet our second design goal we needed a robust reporter of gene expression. We chose a fast-folding, enhanced-fluorescence green fluorescent protein (GFP) variant (*gfp*mut3*) as a reporter as it can be measured via many methods [4, 5].

To meet our third design goal we chose as reference standards a 35bp constitutive promoter (BBa_J23101) that is similar in activity to promoters that have been previously used in a number of engineered biological systems [6-8]. For example, the commonly used P_Ltet-O1_ and P_Lac-O1_ promoters [9] have a measured activity within 10% of the activity of the reference standard promoter.

Meeting our fourth design goal, ensuring that a part characterized in one context behaves equivalently when placed in a different context, is a central challenge in biological engineering [10]. For example, the DNA sequence adjacent to promoters is known to affect their activities [11-13]. The BioBrick assembly standard begins to address the problem of insulating parts from the effects of adjacent sequences by including 8 bp “BioBrick standard junctions” between combined parts, although these junctions can create additional challenges such as causing a frameshift when assembling two protein coding regions.

# Alternate designs for the promoter measurement kit

To explore our design goals we considered an alternate design of the promoter measurement kit. The alternate promoter measurement kit was designed to allow promoters to be easily removed from the testing plasmid (for use in screening promoter libraries) by replacing the XbaI site on the GFP reporter device with a SpeI site. Thus, when a promoter was inserted it could be later removed by digesting with EcoRI and SpeI. However, this design was rejected because it formed a non-BioBrick standard junction (1bp change) between the promoter and RBS that was found to weaken the measured strength of some of the promoters compared to a standard BioBrick control (up to 50% reduction in expression, not shown).

# Transcription start site prediction

We predicted the transcriptional start site for each of the promoters tested using the promoter measurement kit. We predicted the transcriptional start site based on heuristics previously published compilations of *E.coli* sigma70 promoters [14]. The likely site of transcription initiation is within 1bp of the underlined A in each of the sequences below and the yellow highlighting represents the -10 region. All of the J23XXX sequences have identical sequences between the -10 region and the transcriptional start so we only show BBa_J23101 as a representative example of this set.

BBa_J23101:

tttacagctagctcagtcctaggtattatgctagctActagag

BBa_R0011:

aattgtgagcggataacaattgacattgtgagcggataacaagatactgagcacatActagag

BBa_R0040:

tccctatcagtgatagagattgacatccctatcagtgatagagatactgagcactActagag

# Calculating the relationship between PoPS and RPUs

Researchers will often need to convert from RPUs to absolute units as absolute units tie promoter activity to the larger system of related reactions within the cell. To make this conversion a conversion factor should be determined relating PoPS to RPUs under the specific measurement conditions used by the researcher. We have made a best estimate of the conversion factor for PoPS to RPUs under the conditions of the “original” protocol described in Assay of Promoter Collection section in the Methods portion of the main text. However, as noted below (for practical reasons) we make use of estimates for parameter values that were measured previously under different conditions and thus our measure of the PoPS to RPU conversion factor under the “original” protocol measurement conditions should be taken as a rough estimate.

1 RPU is equivalent to the steady-state rate of successful mRNA initiation events per DNA copy of the reference standard promoter BBa_J23101 (
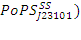
. We can use the equation SM.5 to estimate the activity of BBa_J23101 in PoPS based on the steady-state GFP synthesis rate of cells containing the reference standard construct.


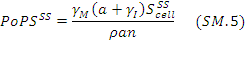


In this case we will need to make estimates of all the rates that we were able to cancel out when making our relative measurement in RPUs (Results). These rates were previously parameterized with an identical reporter mRNA using identical equipment and under similar conditions [15] to the “original” protocol described in the Methods portion of the main text. The GFP maturation rate (
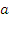
) was found to be 1.8E-3 sec^-1^, the mRNA degradation rate (
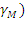
 was found to be 4.8E-3 sec^-1^, and the translation rate from RBS BBa_B0032 was found to be 0.4 proteins sec^-1^ mRNA^-1^. We also measured the growth rate to be 0.51 hr^-1^, and we estimate the copy number of the reference standard plasmid to be approximately 40 copies per cell based on unpublished experiments in the Endy lab with a nearly identical construct (identical except that the promoter was BBa_R0011 rather than BBa_J23101). However, for the copy number measurement, the cells were grown under different conditions (continuous rather than batch culture). Furthermore, constructs with the same origin of replication as the reference standard construct (p15A) have been shown to have copy numbers of 20-30[9] or as low as 14-16 [16], so this parameter should be considered a rough estimation. We used the data described previously (in Results, community-based measurement of promoter collections) of fluorescence and absorbance measured for cells in midexponential growth in a multi-well fluorimeter to estimate GFP synthesis rate:


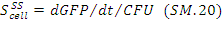


We used the following conversions measured previously in the Endy lab under similar conditions [15] relating background subtracted absorbance (
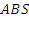
) to colony forming units (
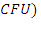
and background subtracted fluorescence (
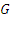
) to molecules of GFP (
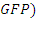
.


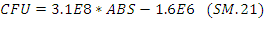


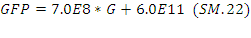


Combining Eqs. SM.20-22, and our measurements of fluorescence and absorbance we find GFP synthesis to be
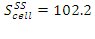
 GFP molecules cell^-1^ sec^-1^. If we combine the rates described above
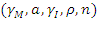
and this measure of GFP synthesis in Eq. SM.5 we find:


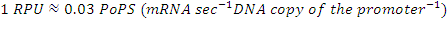


# Parameter sensitivity

To simplifying our model for calculating RPUs from steady-state GFP synthesis rates, we assume the equivalency of many parameters between the reference promoter and the test promoter. In order to test the sensitivity of our RPU calculation to these assumptions of equivalence, we individually varied (increased and decreased) each parameter for the reference promoter across a 100-fold range while holding the corresponding parameter for the test promoter constant. We then determined the error in the RPU calculation by taking the ratio between the predicted RPUs when the parameters were varied to that when they were held equivalent (Supplementary Figure 3). We used the same values for the parameters as were listed above in the section determining the relationship between RPU and PoPS. Our parameter sensitivity analysis yielded:

Zeroth-order relationship

Immature GFP total degradation rate (
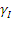
 )

First-order relationship

mRNA degradation rate (
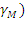


Translation rate of immature GFP from mRNA (
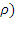


Copy number of the plasmid (
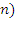


GFP maturation rate (
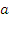
)
Second-order relationship

Growth rate (
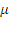
 )

We found that the when the model parameters were varied over a 10-fold range (10-fold lower and higher than the initial parameter values) that the GFP degradation rate had essentially no effect on RPU calculations. Variation in the GFP maturation rate had a low effect with a 2-fold change in RPU due to a 10-fold reduction in GFP maturation rate (an increase in GFP maturation rate had essentially no effect). Variation in the growth rate had a low effect with a 3-fold change in RPU due to a 10-fold increase in growth rate (a 10-fold decrease in growth rate had little effect). Finally, the mRNA active degradation rate, translation rate of immature GFP from mRNA, and copy of number of the plasmid showed a 10-fold change in the parameters yielding a 10-fold change in the calculated RPUs (for both increased and decreased values of the parameters). Thus, for a reasonable (10-fold) region of error in our model parameters these 3 parameters are those that will have the greatest impact on the accuracy of our models.

# Supplementary references

# Supplementary figures

## Supplementary Figure 1 - Example of typical growth curves

Growth curves for three replicates of each of the four promoters used in the inter-laboratory test (BBa_J23113, BBa_J23150, BBa_J23151, BBa_J23102), the reference standard promoter (BBa_J3101), and the negative control (TOP10 with no plasmid). The growth curves suggest that the cells are in exponential growth for about an hour (0.5-1.5 hrs), the vertical dotted lines indicate the region sampled to calculate the steady state GFP synthesis.

## Supplementary Figure 2 - Example of GFP synthesis rate time series

The GFP synthesis rate (d[GFP]/dt / ABS) for three replicates of each of the four promoters used in the inter-laboratory test (BBa_J23113, BBa_J23150, BBa_J23151, BBa_J23102), the reference standard promoter (BBa_J3101), and the negative control (TOP10 with no plasmid). There is a region of steady-state GFP synthesis from approximately 0.5 hrs to 1.25hrs. The vertical dotted lines indicate the region sampled to calculate the steady-state GFP synthesis rate.


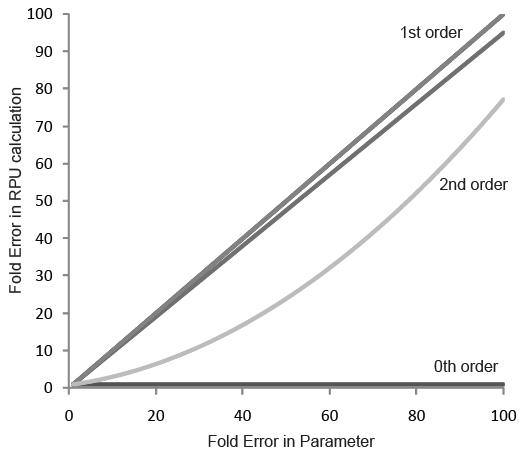


## Supplementary Figure 3 - Parameter sensitivity

A parameter sensitivity analysis was conducted on our model for calculating RPUs by increasing or decreasing the value of each parameter 100-fold. For simplicity we only plotted error as a result of increasing the parameter values (see below). We found that changes in parameters had 0^th^, 1^st^, or 2^nd^ order effects on the calculated RPU value. The 0^th^ order parameter was Immature GFP total degradation rate (
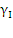
 ). The 1^st^ order relationships were mRNA degradation rate (
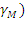
, translation rate of immature GFP from mRNA (
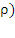
, copy number of the plasmid (
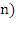
, and GFP maturation rate (
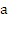
). Although, of these GFP maturation rate only showed a 1^st^ order relationship for decreases in the value of the parameter (it was 0^th^ order otherwise) and mRNA degradation rate only showed a 1^st^ order relationship for increases in the value of the parameter (it was 0^th^ order otherwise). A 2^nd^ order relationship was found for increases in growth rate (
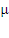
 ) (it was 0^th^ order otherwise).


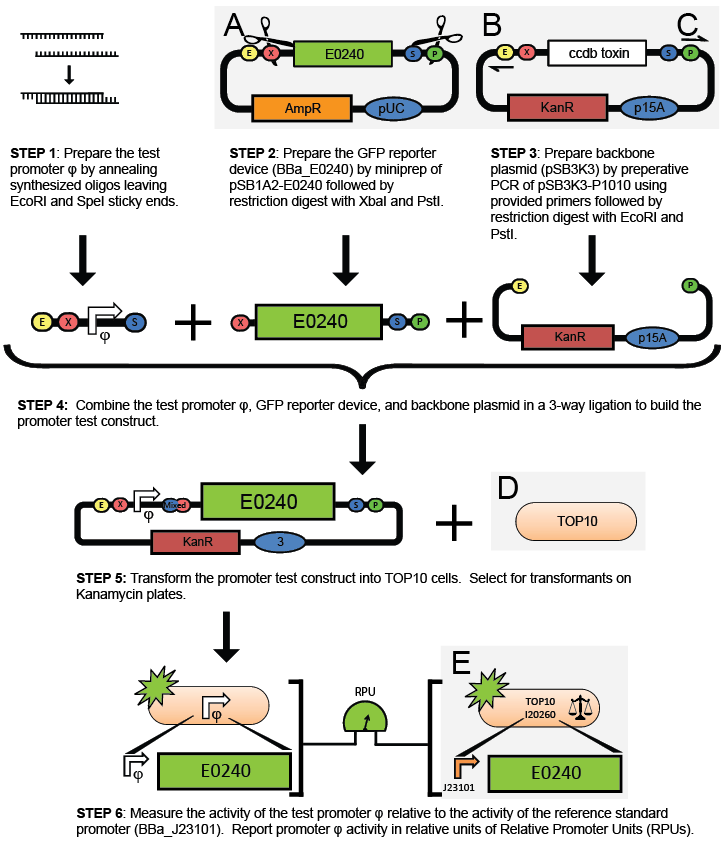


## Supplementary Box 1 - Instructions for inserting a promoter into the promoter measurement kit and measuring the promoter activity in Relative Promoter Units (RPUs).

The components marked A-E and shaded in gray are included with the kit: **(A)** pSB1A2-E0240 is provided as purified DNA. BBa_E0240 is the GFP reporter device (containing an RBS, GFP coding region, and transcription terminator). **(B)** pSB3K3-P1010 is provided as purified DNA to serve as template for a preparative PCR reaction. **(C)** DNA primers are provided for use in a preparative PCR reaction to produce backbone vector (pSB3K3). **(D)** TOP10 cells are provided to serve as the standard characterization and construction strain. **(E)** pSB3K3-I20260 is provided as a plasmid in TOP10. BBa_I20260 contains the promoter reference standard, BBa_J23101, upstream of the GFP measurement device.

**
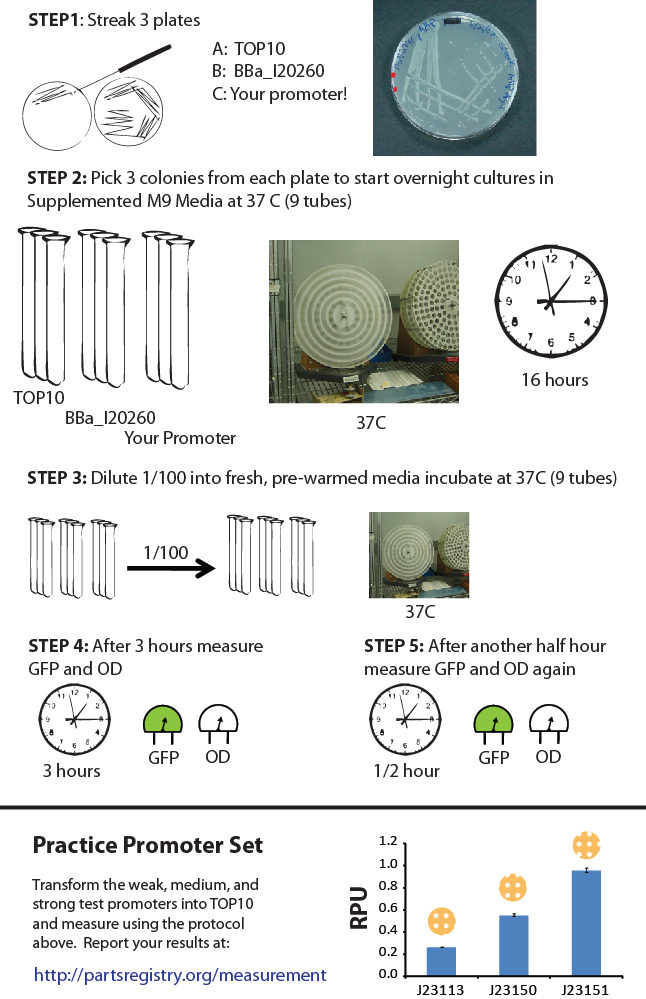
**

## Supplementary Box 2 - Instructions for measuring promoter activity in Relative Promoter Units (RPUs) provided to participants in the International Genetically Engineered Machines (iGEM) competition.

A simple measurement procedure for characterizing promoters in RPUs requiring only two absorbance and bulk fluorescence measurements. The procedure was designed to be simple enough to be carried out by the novice researchers participating in the IGEM competition. A practice promoter set was provided so users could compare their results to those previously measured.

# Supplementary tables

| **Notation** | **Name**  **(BioBrick Part #)** | **Description / Function** |
| --- | --- | --- |
| 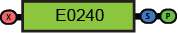 | GFP reporter device  (BBa_E0240) | The **GFP reporter device** contains an RBS, GFP coding region, and transcription terminator. It converts a transcription input into molecules of GFP. |
| 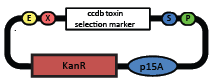 | Backbone plasmid  (pSB3K3-P1010) | The **backbone plasmid** contains an expression cassette for ccdb toxin that serves as a counter-selectable marker as well as the p15A origin of replication and a kanamycin resistance gene. |
| 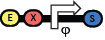 | Test promoter φ | The **test promoter φ** is the user-specified promoter to be measured. |
| 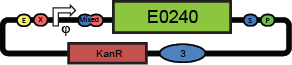 | Promoter test construct | The **promoter test construct** contains the **test promoter** φ upstream of the **GFP reporter device** within the pSB3K3 **backbone plasmid.** The rate of GFP expressed from this construct is used to measure the activity of the test promoter. |
| 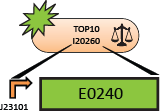 | Reference standard construct  (BBa_I20260) | The **reference standard construct** is identical to the **promoter test construct** except it contains the reference standard promoter (BBa_J23101). The rate of GFP expressed from this construct is used to measure the activity of the reference standard promoter. |
| 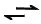 | Preparative primers  (BBa_G1000, BBa_G1001) | The **preparative primers** are used to amplify the pSB3K3 **backbone plasmid**. |
| 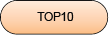 | TOP10  (BBa_V1009) | *E. coli* TOP10 is used as the standard strain for kit measurement experiments. |

## Supplementary Table 1 - Components of promoter measurement kit

The nomenclature listed is used to describe the promoter measurement kit components within the text. The notation for each component serves as a key for the kit instructions (Box1). More detailed information about each component such as the DNA sequence can be found at the Registry of Standard Biological Parts (http://partsregistry.org).

| **Make** | **Laser** | **Excitation Line** | **Emission Filter** |
| --- | --- | --- | --- |
| **BD FACSCalibur** | Argon | 488nm | 530/30 |
| **BD LSR II** | Argon | 488nm | 530/30 |
| **BD LSR II** | Coherent Sapphire | 488nm | 530/30 |
| **Partec CyFlow Space** | Solid state | 488nm | 520/30 |
| **BD FACSAria** | Coherent Sapphire | 488nm | 535/30 |
| **BD LSR II** | Argon | 488nm | 525/50 |
| **BD FACSAria** | Coherent Sapphire | 488nm | 530/30 |

## Supplementary Table 2 - Listing of the flow cytometer equipment used at the seven laboratories that participated in the inter-laboratory variability study.

|  | 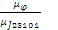(mean) | 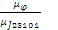(stdev) |
| --- | --- | --- |
| TOP10 | 1.1063 | 0.1413 |
| J23113 | 1.1264 | 0.104 |
| J23150 | 1.21 | 0.1085 |
| J23151 | 1.1875 | 0.1081 |
| J23102 | 1.0985 | 0.0707 |

## Supplementary Table 3 - Growth rates of cells containing the 4 promoter test constructs that were used in the inter-laboratory study measured relative to the reference standard.


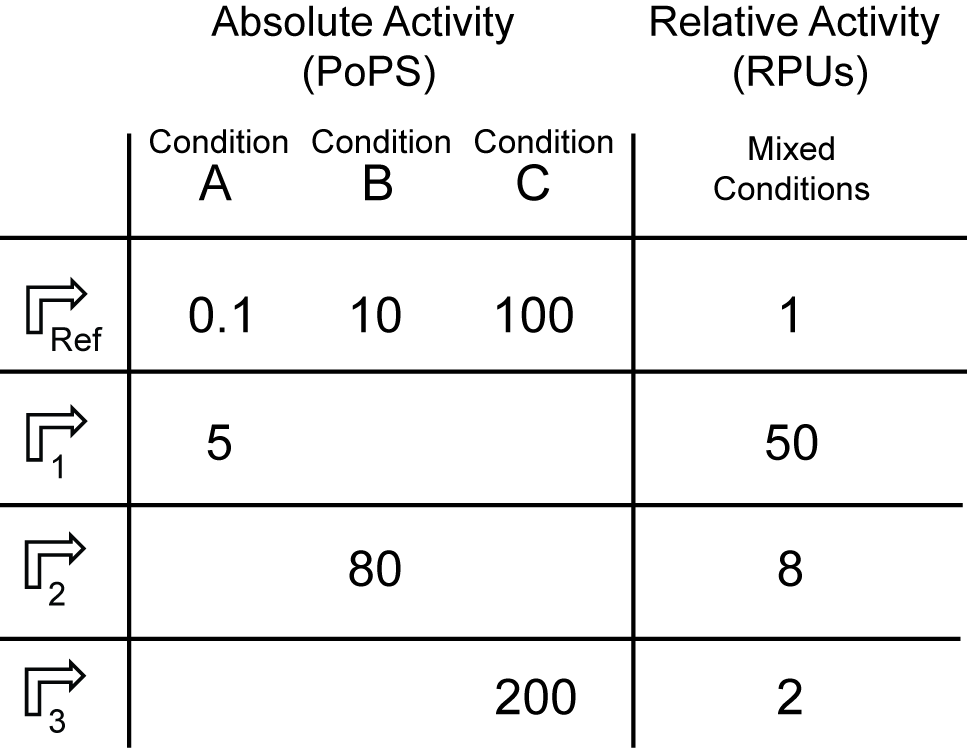


## Supplementary Table 4 - Overview of relative and absolute promoter activities

The table shows example results of characterizing three promoters (1,2, and 3) under three different conditions (A,B, and C). Under each condition the absolute activity of one promoter is measured in PoPS along with the absolute activity of the reference promoter. The ratio of these values (the relative activity of promoter 1, 2 or 3) is then recorded in the fourth column in units of RPUs. Notice that the RPU values provide a relative ranking of the promoters, for instance promoter 1 is 25 times as active as promoter 3, however these RPU values alone provide no information about the absolute activity of any promoter under a particular set of conditions.
